# Multiple cryosauna sessions for post-exercise recovery of delayed onset muscle soreness (DOMS): a randomized control trial

**DOI:** 10.3389/fphys.2023.1253140

**Published:** 2023-09-12

**Authors:** Beata Wolska, Łukasz Domagała, Aleksandra Kisilewicz, Hamidollah Hassanlouei, Piotr Makar, Adam Kawczyński, Sebastian Klich

**Affiliations:** ^1^ Department of Combat Sports, Gdansk University of Physical Education and Sport, Gdansk, Poland; ^2^ Department of Athletics, Gdansk University of Physical Education and Sport, Gdansk, Poland; ^3^ University WSB Merito, Wrocław, Poland; ^4^ Department of Cognitive and Behavioral Sciences and Technology in Sport, Shahid Beheshti University, Teheran, Iran; ^5^ Department of Swimming, Gdansk University of Physical Education and Sport, Gdansk, Poland; ^6^ Department of Biomechanics and Sport Engineering, Gdansk University of Physical Education and Sport, Gdansk, Poland; ^7^ Department of Paralympic Sport, Wrocław University of Health and Sport Sciences, Wrocław, Poland

**Keywords:** cryotherapy, muscle damage, countermovement jump, myotonometry, recovery

## Abstract

The main goal was to investigate the effectiveness of cryosauna in preventing the development of delayed onset muscle soreness and to analyze the regenerative changes within muscles after acute fatigue-induced exercises. Thirty-one volunteers were assigned into two groups: 1) an intervention group that participated in cryostimulation after fatigue-induced exercise protocol (CRYO, *n* = 16) and a control group that performed fatigue-induced exercise protocol, but without any intervention (CONT, *n* = 15). Main outcome measures include at baseline: blood sample testing (leukocyte content, myoglobin concentration, and creatine kinase activity) and muscle stiffness of lower extremity; immediately after (stiffness), and 24-48-72-96 h post-exercise (blood samples and stiffness). Both groups performed an exercise-induced muscle damage protocol based on repeated countermovement jumps (10 sets, 10 repetitions). The CRYO group underwent a cryosauna (temperature: −110°C, time: 1.5 min per session) intervention during four sessions (i.e., immediately after, 24-48-72 h post-exercise). Leukocyte content was significantly greater 24-48-72 h after exercise in CONT, compared with the CRYO group (*p* ≤ 0.05 for all), while creatine kinase activity was greater 24-48-96 h in CONT, compared with the CRYO group (*p* ≤ 0.05 for all). Muscle stiffness increased significantly in rectus femoris, tibialis anterior, and fibula muscle after 48 h post-exercise (*p* ≤ 0.05 for all), as well as in tibialis anterior and fibula after 72 h post-exercise (*p* ≤ 0.05 for all) in the CRYO group. Multiple cryosauna was an effective recovery strategy that reduced blood biomarkers and muscle stiffness after exercise-induced muscle damage. Moreover, the development of delayed onset muscle soreness, expressed by a greater muscle stiffness post-exercise, was attenuated to the first 48 h.

## Introduction

Delayed onset muscle soreness (DOMS) has been defined as the initial type 1) of muscle strain occurring with accompanying pain sensations (Gulick and Kimura, 1996) and characterized by additional stiffness related to increased inflammation (MacIntyre et al., 2001). DOMS appears as early as 8 h after exercise in the form of muscle soreness, peaking between 24 and 48 h after exercises (Kawczyński et al., 2012; Argus et al., 2017), and then gradually relieves and disappears in about 5–7 days due to physiological changes in the muscle fibers. During the last several decades many theories were created to explain the mechanisms of DOMS, however the most recent focused on inflammation and enzyme efflux (Gulick and Kimura, 1996; Cheung et al., 2003). Currently, models integrate different theories together and form a consistent description of different physiological processes (Cheung et al., 2003). The exercise-induced muscle fiber damage may results in disruption of protein structures of a fiber followed by calcium imbalance. During the next few hours neutrophils increases their concentration causing inflammation (Smith, 1991). The increase in creatine kinase (CK), an marker of connective tissue and muscle damage, causes increase in mast cells and histamine within the next 6–12 h. Moreover, macrophages reach the maximal number within 48 h and produce prostaglandin that affects mechanical and chemical stimulation of type III and IV nerve). Finally, these processes may result in an increase of temperature, which might be an additional symptom of inflammation (Cheung et al., 2003). This leads to muscle stiffness and consequently causes pain that can alter the neuromuscular response during movement and impair the athlete’s performance and training program (Hotfiel et al., 2018; Seco-Calvo et al., 2020).

Muscle damage and soreness are common conditions experienced by athletes and individuals who engage in strenuous exercise. Therefore, several interventions have been suggested to prevent the effects of DOMS. Recently, cryotherapy has emerged as a promising method for reducing DOMS and enhancing post-exercise recovery. There are several cold therapy methods used by athletes: cold water immersion (CWI), contrast water therapy (CWT), whole-body cryotherapy (WBC), and cryosauna (Jajtner et al., 2015; Argus et al., 2017; Lombardi et al., 2017; Seco-Calvo et al., 2020; Miranda et al., 2021). Cryotherapy involves exposing the body to cold temperatures to induce physiological responses that promote healing and regeneration by reducing inflammation, increasing blood flow, and, finally, enhancing muscle recovery (Banfi et al., 2009; Pournot et al., 2011; Siqueira et al., 2018). One of the most significant effects of cryogenic temperatures is the analgesic effect connected with influence on the endocrine system, such as increased secretion of *𝛽*-endorphins, and metabolic action manifested by decreased concentrations of histamine and lactate in changed inflammatory tissues. These effects are thought to be mediated by the activation of various cellular and molecular pathways, including the release of anti-inflammatory cytokines and growth factors, as well as the impact of cryogenic temperatures on the prooxidant-antioxidant balance and stabilization of lysosome membranes and subsequent inhibition of release of active enzymes from lysosomes (Rose et al., 2017; Miranda et al., 2021). The physiological mechanism of action of cryogenic temperatures might be related to the effect on the vasomotor response of the small blood vessels in the skin during and after cryostimulation (Olson and Stravino, 1972) and the mechanism of ischemia and reperfusion (Pumtel et al., 2013). Previous studies have demonstrated the beneficial effect of different cryotherapy methods (e.g., ice massage, CWI, CWT, and WBC) on DOMS, especially in the reduction of post-exercise pressure pain threshold (Klich et al., 2018) and blood biomarkers concentration and activity (Eston and Peters, 1999; Goodall et al., 2008; Machado et al., 2017; Siqueira et al., 2018). Moreover, previous studies reported that a multiple-session intervention of cryotherapy during 72–168 h post-exercise may significantly lead to a decrease in creatine kinase, C-reactive protein, and inflammatory cytokines concentration compared to a non-intervention group (Eston and Peters, 1999; Goodall et al., 2008; Siqueira et al., 2018). However, it should be noted that some studies have not shown any beneficial effects on inflammation, muscle soreness, and stiffness after local cryotherapy (Behringer et al., 2018) and CWI (Pinto et al., 2020).

Despite its growing popularity, the effectiveness of cryotherapy and cryosauna for DOMS relief is still a topic of debate in the scientific community. Cryosauna is a type of partial-body cryotherapy that might be used in sports regeneration because of a lower temperature (up to −160°C) at the initial stage compared with WBC. The lower temperature is caused by a direct contact between the medium and the user. Additionally, it should be noted that cryosauna has no vestibule chamber (Piotrowska, 2017). Furthermore, the mechanisms by which cryotherapy exerts its effects on DOMS are not fully understood (Lombardi et al., 2017). There are a few studies that have investigated the effect of a series of whole-body cryotherapy for athletes using a cryo-chamber or cryosauna based on changes in biochemical blood indices and pain evaluation (Hausswirth et al., 2011; Pournot et al., 2011; Kozłowska-Flis et al., 2021).

To our knowledge, no study has addressed the effectiveness of cryosauna in the prevention of DOMS by the determination of changes in the levels of leukocyte content, myoglobin concentration, creatine kinase activity with the quantified measurements of muscle stiffness. Therefore, in the present study, we have focused not only on changes in biochemical blood indices but also on the properties of the muscles themselves, as an increase in muscle stiffness is one of the main symptoms of DOMS, which can increase the risk of injury and reduce exercise capacity. Moreover, our research has been extended to 96 h long, and we looked at biochemical indices and muscle stiffness at 24, 48,72, and 96 h after exercise, when most studies describing the effects of cold therapy after exercise monitored its effectiveness for up to 48 h (Lombardi et al., 2017). Although, as recommended in the literature, all cryotherapy sessions were performed up to 72 h after a counter-movement jumps (CMJ) series due to the duration of the destruction phase of muscle regeneration (Rose et al., 2017). Therefore our goals were to investigate the effectiveness of cryosauna in preventing the development of DOMS and to analyze the regenerative changes within muscles after acute fatigue-induced exercises. We hypothesized that multiple cryosauna sessions would reduce the effect of DOMS within 96 h post-exercise by a decrease of blood biomarkers of muscle damage and optimization of muscle stiffness (Eston and Peters, 1999; Goodall et al., 2008; Point et al., 2018; Siqueira et al., 2018).

## Materials and methods

### Participants

The eligible population of thirty-six healthy and active students who specialized in martial arts volunteered in this study. A detailed description of participant demographics and performance parameters is included in Table 1. All the participants had training experience in martial arts (judo and karate) of 12 ± 4 years and a training duration of 10 ± 2 h per week. The weekly training routine included aerobic, strength, and specific martial arts training. The participants were asked to resign from training and avoid strenuous physical activity 48 h before data collection. Inclusion criteria were: 1) no previous experience with cryostimulation using a cryosauna, 2) no supplementation or prescribe drugs, and 3) recreational lifestyle. The exclusion criteria included: 1) current or prior pain or injury in the lower extremity; 2) prior history of surgery in the lower extremity, and 3) cardiovascular diseases. All participants were fully informed about the procedures, read and signed written informed consent, and agreed to participate in this study.

**TABLE 1:** Participant demographics and performance parameters of mean and standard deviation in both groups.

Variables	Experimental group *n* = 16	Control group *n* = 15
Demographics		
Age [years]	22.1 ± 1.8	21.8 ± 1.6
Body height [cm]	176.3 ± 8.3	175.3 ± 11.5
Body weight [kg]	76.1 ± 17.1	76.2 ± 17.2
Body Mass Index [kg/m^2^]	23.3 ± 3.5	24.2 ± 3.7
Dominant lower extremity		
Right	13	15
Left	3	0
Performance parameters		
Jump height [cm]	32.2 ± 5.4	32.8 ± 5.4
Average force [N]	1350.3 ± 237.4	1035.9 ± 208.2
Relative power [W/kg]	5.2 ± 2.9	5.4 ± 1.5

### Study design

This study design was a randomized, controlled, single-blind trial with repeated measures. Moreover, the design was prepared according to the Consolidated Standards of Reporting Trials (CONSORT) for pragmatic trials. During the randomization, one participant was excluded from this study because have not met the inclusion criteria. The allocation of groups was randomly generated using Research Randomizer v.4. Individual and sequentially numbered cards containing the random assignment were folded in sealed opaque envelopes, and then an external researcher selected the envelope to allocate the participants. Then, the assignment was revealed after baseline data collection to the participant but not to the examiner and rater.

**FIGURE 1:**
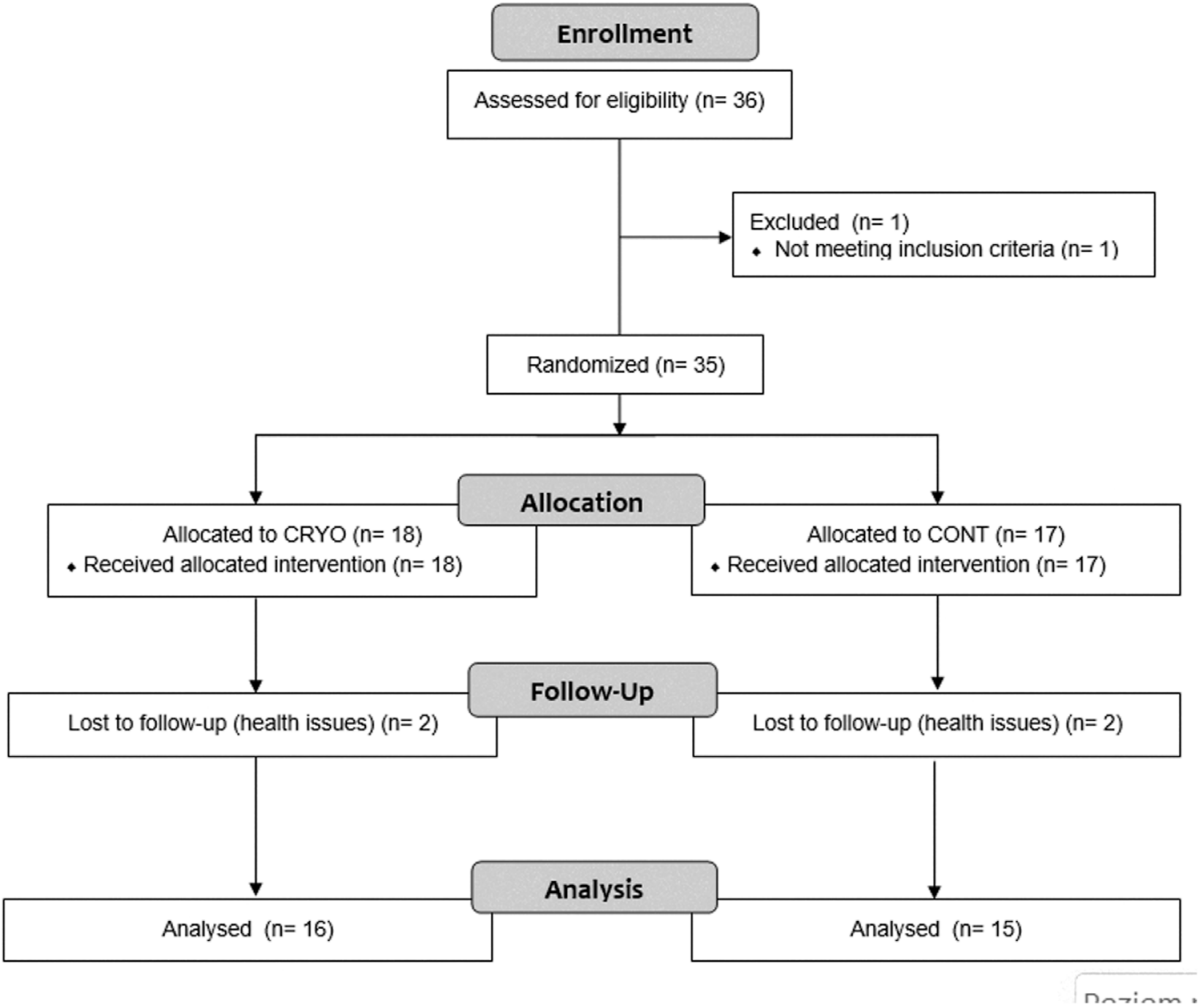
Experimental procedures. Fatigue-induced protocol consisting of CMJ (10 sets for 10 repetitions). After the fatigue exercises, only CRYO underwent a cryosauna intervention including entry immediately after, 24-48-72 h post-exercise. Measure outcomes at baseline: blood sample testing (leukocyte content, myoglobin concentration, and creatine kinase activity) and muscle stiffness of the dominant lower extremity; immediately after (stiffness), and 24-48-72-96 h post-exercise (blood samples and stiffness).

Participants were assigned into two groups: 1) an intervention group that participated in cryostimulation after fatigue-induced exercise protocol (CRYO, *n* = 18), and a control group performing fatigue-induced exercise protocol, but without any intervention (CONT, *n* = 17). However, during the process, four participants (two in CRYO and two in the CONT group) resigned from participation in the further part of the study for health reasons. Finally, the analysis was performed on thirty-one participants, including sixteen in CRYO and fifteen in the CONT group (Figure 1).

A familiarization session was performed 1 week before the main experiment, where all participants were informed about the study design and signed the informed consent. Furthermore, during this session, participants were instructed to exclude criteria, avoid strenuous exercise during the study, and follow safety rules in cryosauna. Next, participants were instructed to a proper counter-movement jumps (CMJ) using a Kistler force plate (model: 9286BA; Winterthur, Switzerland). During the test, all participants performed two CMJs from a bounce with both feet and a standing position with their hands placed on their hips. This performance measure was used for the detailed description.

The G*Power software (version 3.1.9.2; Kiel University, Kiel, Germany) was used to estimate the required sample size. A sample size estimated with a repeated measure ANOVA within and between factors, an α of 0.05, set a minimum expected effect size (Cohen’s *f*) of 0.25, *β* of 0.95, and correlation among repeated measures of 0.5. The number of measurements for blood samples was five, while for stiffness was six. The procedure included a minimum number of 32 participants required for blood sample testing, however 28 participants for stiffness measurements. Therefore, four extra participants were recruited to account for potential drop-out.

### Experimental protocol

Research procedures and data collection were conducted in the Exercise Physiology Laboratory at Gdansk University of Physical Education and Sport. The laboratory has special facilities for blood sample taking and testing and monitoring exercise outcomes. The room temperature and humidity are 21°C (69.8°F) and 60%, respectively. The order of measurements included at baseline: blood sample testing (leukocyte content, myoglobin concentration, and creatine kinase activity) and muscle stiffness of the dominant lower extremity; immediately after (stiffness), and 24-48-72-96 h post-exercise (blood samples and stiffness). The examiners responsible for blood sample tests and stiffness were blinded and thus were not informed about the order of participants and being assigned to one of the groups. After baseline measurements, all participants performed 10-min warm-up exercises based on global exercises of the lower extremity and trunk joints and muscles. Following a 5-min passive rest, each participant performed repeated CMJ jumps (10 sets of 10 repetitions within a 1-min rest between sets) (Kons et al., 2020).

### Intervention

**FIGURE 2:**
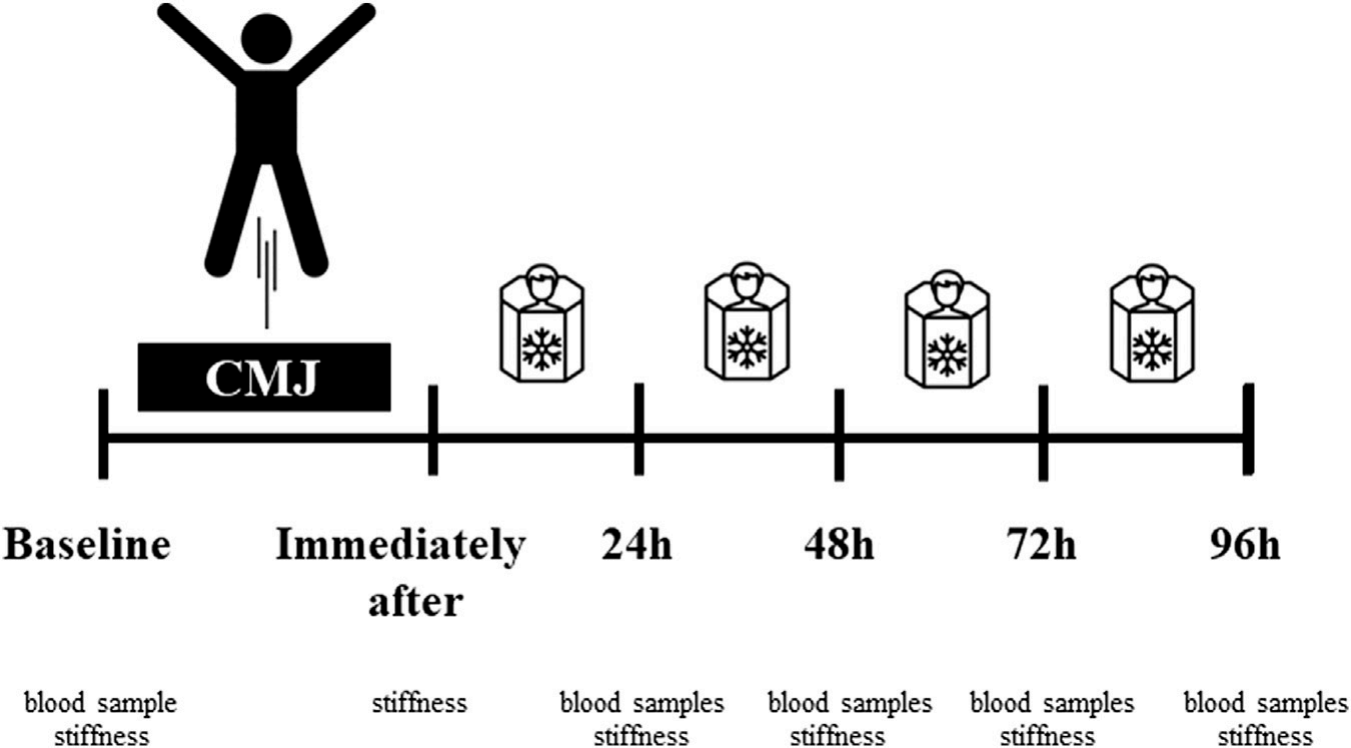
Experimental procedures. Fatigue-induced protocol consisting of CMJ (10 sets for 10 repetitions). After the fatigue exercises, only CRYO underwent a cryosauna intervention including entry immediately after, 24-48-72 h post-exercise. Measure outcomes at baseline: blood sample testing (leukocyte content, myoglobin concentration, and creatine kinase activity) and muscle stiffness of the dominant lower extremity; immediately after (stiffness), and 24-48-72-96 h post-exercise (blood samples and stiffness).

After the fatigue exercises, only CRYO participated in a cryostimulation intervention during four sessions (i.e., immediately after, 24-48-72 h post-exercise) of cryo-cabin where the head was not exposed (cryosauna) (JUKA Standards: EN ISO 9001:2009–EC Certificate of Conformity to Directive 93/42/EEC/Annex VI on Medical Devices– ISO 13485: 2003). All sessions took place between 10–11 a.m. from Monday to Thursday under the supervision of a qualified staff member. Each session lasted 1.5 min. Before entering the cryosauna, participants had their blood pressure measured. The temperature in the cryosauna was −110°C. During this intervention, participants wore only shorts, socks, gloves, and hats (Figure 2).

### Outcomes

#### Blood biomarkers of muscle damage

Blood samples were collected by standard intravenous puncture by a qualified nurse before the fatigue procedure and also 24-48-72-96 h post-exercise. The test consisted of drawing 5 mL of blood from a vein (in the ulnar fossa) into a tube containing EDTA (Becton Dickinson and Co., Franklin Lakes, NJ, United States). Blood samples were centrifuged twice (1,600× *g*, 10 min) within 2 h after collection (prior stored at 4°C). Clear plasma samples were transferred to fresh Eppendorf tubes and stored at −80°C until further analysis. For the data analysis, leukocyte content [10^9^/L], myoglobin concentration [ng/mL], and creatine kinase activity [U/L] were measured. Leukocyte content was analyzed using an automated hematologic analyzer (SYSMEX XN 2000; SYSMEX Corporation, Kobe, Japan). This measurement was based on fluorescence flow cytometry using a semiconductor laser that measures cells at a wavelength of 663 nm. The measurement detailed characteristics, i.e., analysis range: 0.0-440 G/L and coefficient of variation (95% confidence) when analyzing peripheral blood −3.0% or less (4.00 × 10^3^/ul or less). Enzyme-linked immunosorbent assays were used to assess changes in myoglobin concentration (Human Myoglobin Matched Antibody Pair Kit AB215407m Cambridge, United Kingdom) and creatine kinase activity was analyzed with an EC 2.7.3.2 Assay Kit (Biosystems, Barcelona, Spain). Normal value range for leukocytes concent was 4.5–11.0 × 10^9^/L; myoglobin concentration was 25–72 ng/mL, as well as creatine kinase activity was 30–135 [U/L] (Chernecky and Berger, 2013).

#### Muscle stiffness

A hand-held myotonometer (MyotonPro, Myoton Ltd., Estonia) was used to measure the stiffness of the lower extremity muscles. The myotonometer measurements were performed on the dominant lower limb (right) at 23 reference points, covering the muscles of the anterior thigh (points 1–7): posterior thigh (points 8–15), shin muscles (points 16–18), and calf muscles (points 19–23). The specific muscles analyzed in this study were as follows: rectus femoris (points 1–2), tensor fasciae latae (point 3); vastus lateralis (points 4–6); vastus medialis (point 7); external extensor muscles (points 8–11); internal extensor muscles (points 12–15); tibialis anterior (point 16); peroneus (points 17–18); external gastrocnemius muscles (points 19–20); internal gastrocnemius (points 21–22); and soleus muscles (point 23) (Klich et al., 2018; Klich et al., 2020). In this study, three measurements were collected at each reference point, and the average was calculated. The measurement for one reference point took about 5 s, while the total test time was less than 2 min.

The relative intra-reliability revealed excellent reliability and acceptable measurement error in a pilot study of *n* = 15 participants for a single point (no. 2) of the quadriceps muscle stiffness (ICC_2, 1_ = 0.91). The absolute reliability showed SEM was 12 N/m, while MDC_90%_ was 27 N/m.

#### Statistical analysis

SPSS statistical software (version 18., SPSS Inc., Chicago, Illinois, United States) was used for all statistical tests and data analysis. Mean values ±standard deviation with confidence interval (CI 95%) were reported in this study. The normality of the data distribution was applied through the Shapiro–Wilk tests, while homogeneity of variance was analyzed by Levene’s test. The analyzed data were normally distributed for all parameters, while the variances for all parameters were equal. A two-way repeated measure analysis of variance (RM-ANOVA) with *Time* (baseline, 24-48-72-96 h post-exercise) and *Group* (CRYO and CONT) was conducted for blood fatigue tests (leukocyte content, myoglobin concentration, and creatine kinase activity). Additionally, a three-way RMANOVA with *Time* (baseline, post-exercise, and 24-48-72-96 h post-exercise), *Group* (CRYO and CONT), and *Muscle* (RF-TFL-VL-VM-TIB-FIB-HAM-CALF) were conducted for muscle stiffness. The Bonferroni adjustment for multiple comparisons was used for *post hoc* tests (*p* = 0.05) if a significant interaction between variables was found. The effect size was estimated using partial eta square (*η* ^2^), classified as small (*η* ^2^ = .0.01), medium (*η* ^2^ = .06), or large (*η* ^2^ = .14) (Cohen, 1988). A *p*-value ≤0.05 was considered significant.

## Results

### Blood biomarkers of muscle damage

**FIGURE 3:**
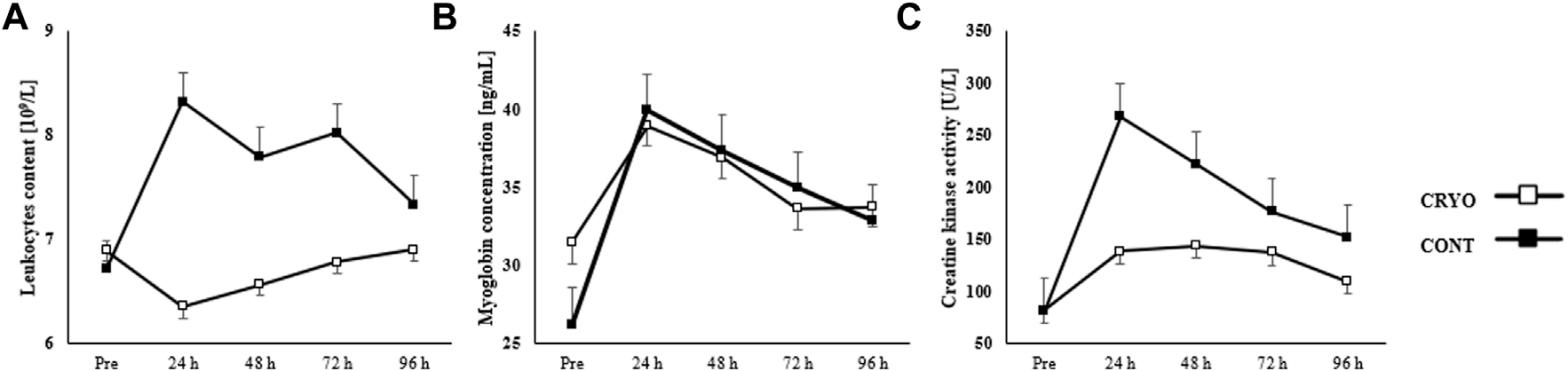
Mean ± SD from pre-test (Pre) and time course (24-48-72-96 h) values for the experimental (CRYO) and control (CON) group for **(A)** leukocyte content, **(B)** myoglobin concentration, and **(C)** serum kinase creatinine (CK) activity after fatigue-induced CMJ protocol. Significant differences *- within-group differences between Pre and 24-48-72-96 h after exercise, and **- between-group differences between CRYO and CONT (*p* ≤ 0.05).

Figure 3 showed the mean ± SD of blood sample tests, including leukocyte content, myoglobin concentration, and creatine kinase activity at baseline, 24-48-72-96-h after fatigue-induced jumping protocol in both groups (experimental and control). The two-way RM-ANOVA revealed a statistically significant main effect of Group (F_1,29_ = 13.2, *p* = 0.001, *η* ^2^ = 0.31) for leukocyte content; Time (F_4,116_ = 7.4, *p* ≤ 0.001, *η* ^2^ = 0.20) for myoglobin concentration; as well as Time (F_4,116_ = 9.6, *p* ≤ 0.001, *η* ^2^ = 0.25) and Group (F_1,29_ = 18.9, *p* = 0.001, *η* ^2^ = 0.49) for creatine kinase activity. The analysis revealed a statistically significant interaction effect between Time and Group (F_4,116_ = 3.0, *p* = 0.025, *η* ^2^ = 0.10) for leukocyte content and (F_4,116_ = 2.6, *p* = 0.04, *η* ^2^ = 0.08) for creatine kinase activity.

Leukocyte content increased significantly 24 h after CMJ protocol (*p* = 0.008), while myoglobin concentration after 24-48-72 h (*p* = 0.001 for all), and creatine kinase activity after 24-48-72-96 h post-exercise (*p* ≤ 0.001 for all) in CONT group. However, we found a significant increase in myoglobin concentration after 24 h (*p* = 0.001) in the CRYO group. Finally, the leukocyte content was significantly greater 24-48-72 h after exercise in CONT, compared with the CRYO group (*p* ≤ 0.05 for all), while creatine kinase activity was greater 24-48-96 h in CONT, compared with the CRYO group (*p* ≤ 0.05 for all) (Figure 3).

### Muscle stiffness

**FIGURE 4:**
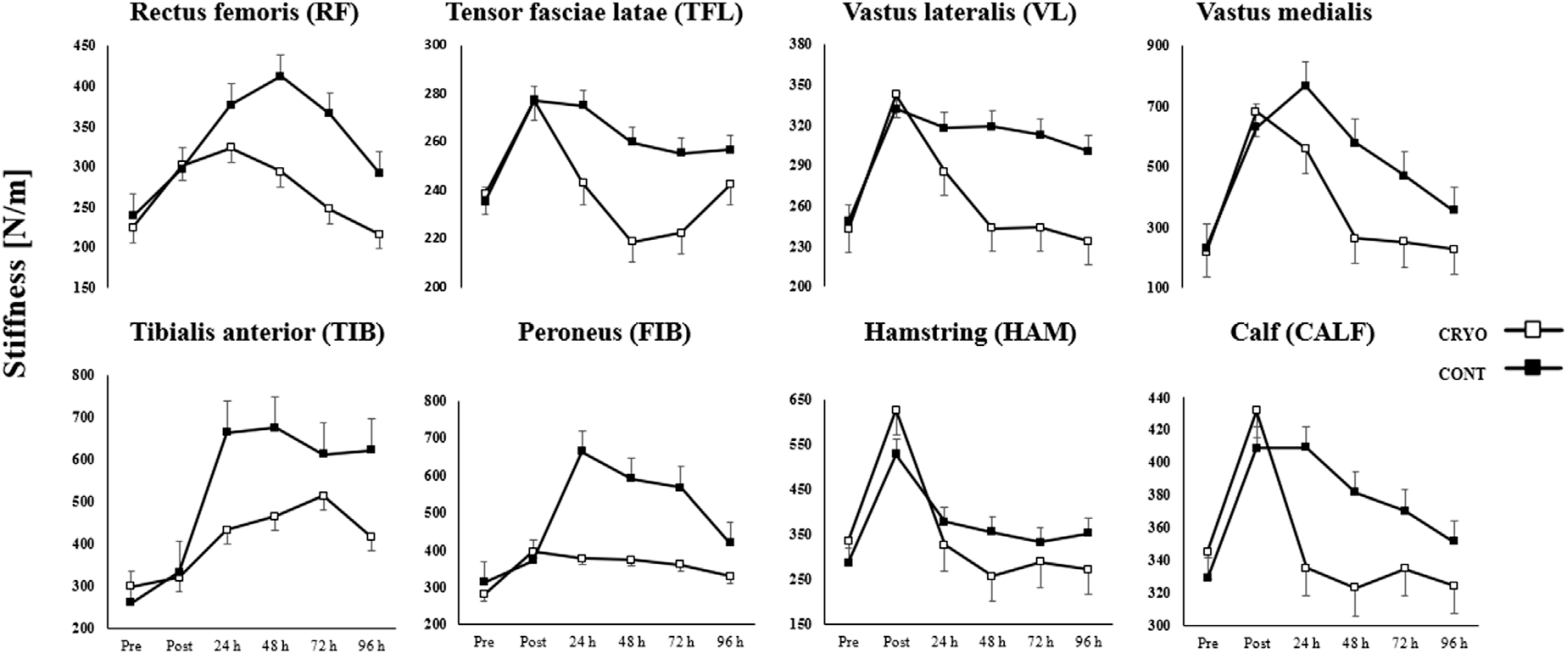
Mean ± SD from pre-test (Pre), immediately after (Post) and time course (24-48-72-96 h) values for the experimental (CRYO) and control (CON) group for lower extremity muscle stiffness: rectus femoris (RF), tensor fasciae latae (TFL); vastus lateralis (VL); vastus medialis (VM); tibialis anterior (TIB); peroneus (FIB); hamstring (HAM); and calf (CALF) muscles after fatigue-induced CMJ protocol. Significant differences *- within-group differences between Pre, Post, and 24-48-72–96 h after exercise, and **- between-group differences between CRYO and CONT (*p* ≤ 0.05).

Figure 4 demonstrates the mean ± SD of the lower extremity muscle stiffness at baseline, post-exercise, and 24-48-72-96-h after CMJ protocol in the experimental and control group. The three-way RM-ANOVA revealed a statistically significant main effect of *Time* (F_5,1080_ = 257.1, *p* ≤ 0.001, *η* ^2^ = 0.54), *Group* (F_1,216_ = 430.0, *p* ≤ 0.001, *η* ^2^ = 0.67) and *Muscle* (F_35,1080_ = 7.3, *p* ≤ 0.001, *η* ^2^ = 0.19). The analysis revealed a statistically significant interaction effect between *Time*, *Group,* and *Muscle* (*F*_35,1080_ = 7.3, *p* ≤ 0.001, *η* ^2^ = 0.19).

Muscle stiffness increased significantly in all examined muscles (except TFL) post-exercise (*p* ≤ 0.05 for all) and in RF, VM, TIB, and FIB after 24 h post-exercise (*p* ≤ 0.05 for all) in the CRYO group. Moreover, an increase was found in RF, TIB, and FIB after 48 h post-exercise (*p* ≤ 0.05 for all), as well as in TIB and FIB after 72 h post-exercise (*p* ≤ 0.05 for all) in the CRYO group. The TIB and FIB muscle stiffness increased after 96 h post-exercise (*p* ≤ 0.05 for all) in the CRYO group (Figure 4).

Moreover, only HAM muscle stiffness decreased after 48 and 96 h post-exercise (*p* ≤ 0.05 for both). For the CON group, muscle stiffness increased significantly in VM and TIB after all time courses (i.e., post-exercise, 24-48-72-96 h) (*p* ≤ 0.05 for both), while VL and FIB expect 96 h after CMJ protocol (*p* ≤ 0.05 for both). The HAM and CALF muscle stiffness increased significantly post-exercise (*p* = 0.001 for both) and after 24 h (*p* ≤ 0.05 for both), while RF post-exercise (*p* = 0.015) (Figure 4).

An increase was found in HAM after 48 h post-exercise (*p* = 0.001), as well as in RF, VL, and FIB after 72 h post-exercise (*p* ≤ 0.05 for all) in the CONT group. Finally, VM, TIB, FIB, and HAM muscle stiffness increased after 96 h post-exercise (*p* ≤ 0.05 for all) in the CONT group. The *post hoc* analysis also showed a significantly greater stiffness post-exercise and lower after 48 h of CMJ protocol in HAM, compared with CALF (*p* = 0.001 and *p* = 0.002, respectively), while greater stiffness post-exercise in HAM, compared with RF and VL (*p* ≤ 0.001 for both) in CRYO group (Figure 4).

However, a lower stiffness post-exercise and after 24 h was found in HAM and RF, compared with VM (*p* ≤ 0.001 for all) in the CRYO group. For the CON group, a greater stiffness was observed post-exercise, while a lower stiffness after 24, 72 h post-exercise in HAM, compared with CALF (*p* ≤ 0.001 for all). Moreover, a greater stiffness was found in VM post-exercise, after 24 and 48 h, compared with RF, VL, and HAM (*p* ≤ 0.05 for all) in the CONT group. Finally, the stiffness of RF, VL, VM, TIB, FIB, and HAM was significantly greater post-exercise in CONT compared with the CRYO group (*p* ≤ 0.05 for all) (Figure 4).

## Discussion

This study investigated the effectiveness of multiple cryosauna sessions on the reduction of DOMS in lower extremity muscles. Our results showed a recovery effect in muscle stiffness after exercise-induced muscle damage. This finding may indicate changes in blood biomarkers of muscle damage and muscle stiffness. Moreover, we have confirmed our hypothesis that multiple cryosauna during the 96 h post-exercise would reduce the post-exercise DOMS, expressed by muscle damage indicators, and support the recovery effect of specific kinds of cryotherapy sessions. Specifically, we observed a statistically significant increase in blood biomarkers of muscle damage after 24 h post-exercise (myoglobin concentration) in the CRYO group. However, a significant increase in leukocyte content after 24 h, myoglobin concentration after 72 h, and creatine kinase activity after 96 h post-exercise were found in the CON group. Muscle stiffness showed a significant increase in stiffness of VM after 24 h post-exercise; RF after 48 h post-exercise; FIB after 72 h post-exercise, as well as TIB after 96 h post-exercise, however a decrease in muscle stiffness was found in HAM after 48 and 96 h post-exercise. In the COM group, we observed a significant increase in muscle stiffness of CALF after 24 h post-exercise; HAM after 48 h post-exercise; RF and VL after 72 h post-exercise, as well as VM, FIB, and TIB after all time courses (up to 96 h post-exercise).

The current study design was constructed to apply four cryosauna sessions during a 96 h post-exercise period. To the best of our knowledge, this is the first study that used cryosauna to evaluate DOMS following exercise-induced muscle damage. Previous studies used a similar intervention based on four cryotherapy sessions (using cold water immersion–CWI) during 72–168 h post-exercise (Eston and Peters, 1999; Goodall et al., 2008; Machado et al., 2017; Siqueira et al., 2018), however, Vaile et al. (2008) compared three different interventions, e.g., cold water immersion, hot water immersion, and contrast water therapy. The results of our study have demonstrated that multiple cryosauna sessions may influence the recovery of lower extremity muscles by reducing the blood biomarkers and muscle stiffness after exercise-induced muscle damage. The intervention group (CRYO) was characterized by significantly lower blood biomarkers after 24–72 h post-exercise (leukocyte content) and 24, 48, and 96 h post-exercise (creatine kinase activity), compared to the CON group. Moreover, in VL, VM, HAM, and CALF, we observed a decrease in muscle stiffness post-exercise (after 48 h) compared to baseline; however, only in HAM, the decrease was statistically significant. In previous studies, authors have not found CWI as much effective in improving the recovery process by reducing post-exercise blood biomarkers concentration and increasing muscle functional performance (e.g., range of motion and strength) (Eston and Peters, 1999; Goodall et al., 2008; Machado et al., 2017; Siqueira et al., 2018). Probably, the reason for this low effectiveness might be related to water temperature and the duration of a single session. Fonda and Sarabon (2013) reported that whole-body cryotherapy with temperatures at −140 and −195°C (with a 3-min duration) might reduce DOMS. In our study, we used multiple cryosauna recovery strategies (four sessions), including a 1.5 min duration of cryostimulation at −110°C.

### Blood biomarkers of muscle damage

The observations from our study have confirmed our expectations about the development of DOMS post-exercise. We observed an increase in blood biomarkers concentration after 24 h post-exercise, while in the lower extremity muscles stiffness immediately after, as well as in some cases a continuous increase until 24 h post-exercise in the CON group. However, the stiffness of RF increased to the first 48 h post-exercise in the CON group. Muscle stiffness in most of the analyzed muscles increased immediately after exercise and decreased after the first session of cryosauna.

Previous studies have shown an ineffective role of a single cryotherapy session on creatine kinase activity and C-reactive protein concentration in the blood after exercise (Lombardi et al., 2017). The use of multiple cryostimulation sessions after exercise or training may decrease creatine kinase activity (30%–40%) (Wozniak et al., 2007; Banfi et al., 2009). Siqueira et al. (2018) investigated other inflammatory blood biomarkers, e.g., interleukin (IL)-6, IL-1a, IL-10, and tumor necrosis factor (TNF)-α. Multiple CWI sessions after exercise-induced muscle damage showed a decrease in these blood biomarkers after 72 h post-exercise. In contrast to the CON group, the tendency of increase after 24 h post-exercise in creatine kinase activity was consistent with the tendency of muscle stiffness. According to previous studies that investigated the effect of multiple CWI on reduce of blood biomarkers, we observed also a decrease in creatine kinase activity after the first cryostimulation. Eston and Peters (1999) demonstrated a decrease in creatine kinase activity after 48 and 72 h post-exercise (eccentric exercise on the elbow flexors), while Vaile et al. (2008) observed after 24 and 72 h post-exercise (eccentric program for lower extremities). In contrast, Siqueira et al. (2018) have not observed a significant decrease in creatine kinase activity post-exercise (drop jump protocol), but only after 168 h post-exercise. In our study, we used exercise-induced muscle damage consisting of CMJ jumps. It should be noted that some studies, e.g., Eston and Peters (1999), investigated DOMS after upper extremity exercises, which might be more susceptible to damage. Finally, the effect of cryotherapy or CWI on leukocyte content and myoglobin concentration have not been evaluated for multiple cryostimulation session post-exercise. Stacey et al. (2010) did not find any positive effect of repeated cycling bouts on leukocyte content after CWI, as well as Krueger et al. (2020) found no reduction in myoglobin concentration after whole-body cryotherapy (at −110°C) followed by high-intensity intermittent exercise. Rodenburg et al. (1995) reported acute biochemical outcomes in creatine kinase activity and myoglobin concentration after eccentric exercise. This study showed an increase in creatin kinase activity after 24 h post-exercise, however, myoglobin concentration increased after 1 h post-exercise. These observations may suggest that myoglobin concentration is increasing immediately after exercise.

### Muscle stiffness

The evaluation of muscle stiffness after exercise-induced muscle damage was shown for the first time in our study. As we expected, muscle stiffness has changed in muscles mainly involved in CMJ. During the stretch-shortening cycle in CMJ, mostly vastus lateralis and medialis, hamstring, and calf muscles are activated (Donohue, 2021). As expected, these muscles, as well as the tibialis anterior and peroneus, reached a higher stiffness immediately after repeated CMJ protocol. For all tested muscles there was a significant decrease in stiffness in all timepoints, for the CRYO group in relation to CON group. The effectiveness of multiple cryosauna on the reduction of muscle stiffness might be related to the alterations in blood biomarkers concentration, which is also in line with the results of our research. The underlining mechanism could be due to the transfer of creatine kinase in the interstitial fluid and the lymphatic system to the bloodstream (Clarkson and Hubal, 2002). Previous studies demonstrated a reduction in muscle and muscle-tendon stiffness after stretch-shortening cycle exercises (Pousson et a., 1991; Avela and Komi, 1998; Toumi et al., 2006; Kubo and Ikebukuro, 2019) using different biomechanical evaluation (surface electromyography), however, there is a lack of use devices based on mechanical properties (myotonometry and elastography). The eccentric phase of stretch-shortening cycle exercise may cause alterations due to muscle damage. According to Harrison and Gaffney (2004), mechanisms responsible for the reduction of muscle stiffness during the stretch-shortening cycle are unknown. However, eccentric exercises may cause DOMS by provoking muscle damage and increasing pain (Kubo and Ikebukuro, 2019), thus the mechanisms theoretically might be related to mechanical and/or metabolic responses (Harrison and Gaffney, 2004). Yanagisawa et al. (2015) considered an increase in creatine kinase activity with alterations in muscle mechanical properties, which is consistent with the results of our study. In general we showed a significant decrease in the level of muscle stiffness, corresponding to a decrease in the level of creatine kinase activity for parallel time points for the CRYO group with respect to the control group.

### Acute mechanisms of DOMS

The acute effect of exercise-induced muscle damage is related to the inflammation process. Acute inflammation requires vascular and cellular response due to vasoconstriction during the initial phase (approximately between 5 and 10 min) and followed by vasodilation and vascular permeability. However, the cellular response uses neutrophils and monocytes. During the next 4 h post-exercise neutrophils increase their concentration and next decreasing concentration, however, monocytes may migrate within the next couple hours. Moreover, the monocytes transfer from blood to tissue during 48 h, increasing their concentration because of an additional transport of plasma proteins to the tissue (Smith, 1991; Stozer et al., 2020). The acute increase in muscle stiffness might be related to alterations in extracellular water volume (Yanagisawa et al., 2015).

### Limitations

Some of the potential limitations should be pointed out to consider in future studies. First, we recruited a group of active participants specialized in martial arts. Future studies should include semi-profession and/or professional athletes to indicate the level of development of DOMS and the effectiveness of recovery strategies. Second, future studies should also include other indicators, such as muscle strength, power and morphological properties assessed by ultrasonography imaging. Third, when calculating the number of participants in future trials, more participants may be required than in the original calculation due to the higher number of potential withdrawals due to blood samples testing and for health reasons.

## Conclusion

Multiple cryosauna was an effective recovery strategy that resulted in a reduction of blood biomarkers of muscle damage (especially creatine kinase activity) and muscle stiffness after repeated CMJ protocol that led to exercise-induced muscle damage. Moreover, the development of DOMS, expressed by greater muscle stiffness post-exercise, was attenuated to the first 48 h. Thus, these multiple recovery strategies should be recommended for sports medicine professionals, such as physical therapists, athletic trainers, and strength and conditioning coaches, to apply after training, competition, and matches.

## Data Availability

The raw data supporting the conclusion of this article will be made available by the authors, without undue reservation.
